# Supplementary material for: Evaluation of Photocatalytic Performance of Nano-Sized Sr0.9La0.1TiO3 and Sr0.25Ca0.25Na0.25Pr0.25TiO3 Ceramic Powders for Water Purification
Source: Nanomaterials (Basel). 2022 Nov 25;12(23):4193. doi: 10.3390/nano12234193 (PMC9736647; doi:10.3390/nano12234193)
Supplement: Supplementary file 1 [file nanomaterials-12-04193-s001.zip › nanomaterials-2017918-supplementary.pdf]

SUPPLEMENTARY MATERIALS

Evaluation of Photocatalytic Performance of $Sr_{0.9}La_{0.1}TiO_3$ and $Sr_{0.25}Ca_{0.25}Na_{0.25}Pr_{0.25}TiO_3$ Nano-Sized Ceramic Powders for Water Purification

Aleksandra Jovanoski Kostić [1], Nikola Kanas [2,*], Vladimir Rajić [3], Annu Sharma [4], Subramshu S. Bhattacharya [4], Stevan Armaković [5,6], Maria M. Savanović [1,6] and Sanja J. Armaković [1,6,*]

[1] University of Novi Sad, Faculty of Sciences, Department of Chemistry, Biochemistry and Environmental Protection, Novi Sad, Serbia
[2] Institute BioSense, University of Novi Sad, Serbia
[3] INS Vinča, Department of Atomic Physics, University of Belgrade, Serbia
[4] Nanofunctional Materials Technology Centre, Department of MME, IIT Madras, India
[5] University of Novi Sad, Faculty of Sciences, Department of Physics, Novi Sad, Serbia
[6] Association for the International Development of Academic and Scientific Collaboration (AIDASCO), Novi Sad, Serbia
* Correspondence: nikola.kanas@biosense.rs (N.K.); sanja.armakovic@dh.uns.ac.rs (S.J.A.);
Tel.: +381 21 485 2137 (N.K.); +381 21 485 2754 (S.J.A.)

Materials and Methods

Synthesis of Materials

Polycrystalline SLTO was synthesized by spray pyrolysis of aqueous nitric solutions, standardized by CerPoTech AS (Trondheim, Norway). The synthesis procedure is the same as for all the oxide ceramic powders that CerPoTech produces (https://www.cerpotech.com/products/product-portfolio).

Degradation procedures

Direct photolysis was carried out under SSI, UV-LED, and UV in a cell made of Pyrex glass (total volume of ca. 40 mL, liquid layer thickness 35 mm) with a plain window on which the light beam was focused. The cell was equipped with a magnetic stirring bar and water circulating jacket. Photodegradation was carried out under SSI, UV-LED, and UV in a cell made of Pyrex glass (total volume of ca. 40 mL, liquid layer thickness 35 mm) with a plain window on which the light beam was focused. The cell had a magnetic stirring bar and water circulating jacket.

Analytical procedures

UFLC–PDA analysis. The suspensions were filtered through Millipore (Millex-GV, 0.22 μm) membrane filters. The lack of adsorption of pindolol on the filters was preliminarily checked. Afterward, a 10 μL sample was injected and analyzed using a Shimadzu UFLC–PDA equipped with an Eclipse XDB-C18 column (150 mm × 4.6 mm i.d., particle size 5 μm, 30 °C). The UV/vis PDA detector was set at 217 nm (wavelength of pindolol's maximum absorption). To shorten the time necessary for the UFLC–PDA analysis and to improve the efficiency of peak separation, a gradient analysis was used instead of an isocratic analysis. The mobile phase (flow rate 0.8 mL/min) was a mixture of ACN and water (the latter acidified with 0.1% H_3PO_4) with the following gradient: 15% ACN at 0 min, which was increased to 30% ACN after 5 min, following which 30% ACN was constant for 5 min; post time was 2 min.

COD Measurements. The calibration curve was obtained using HOOCC6H4COOK as a standard solution, and the *r* was 0.99 (Fig. S1). Absorbance was recorded on a double-beam T80+UV-Vis Spectrometer (UK) at a fixed slit width (2 nm) using a 1 cm quartz cell and computer-loaded UV Win 5 data software. The evolution of absorbance of the digested solution was recorded at 600 nm.

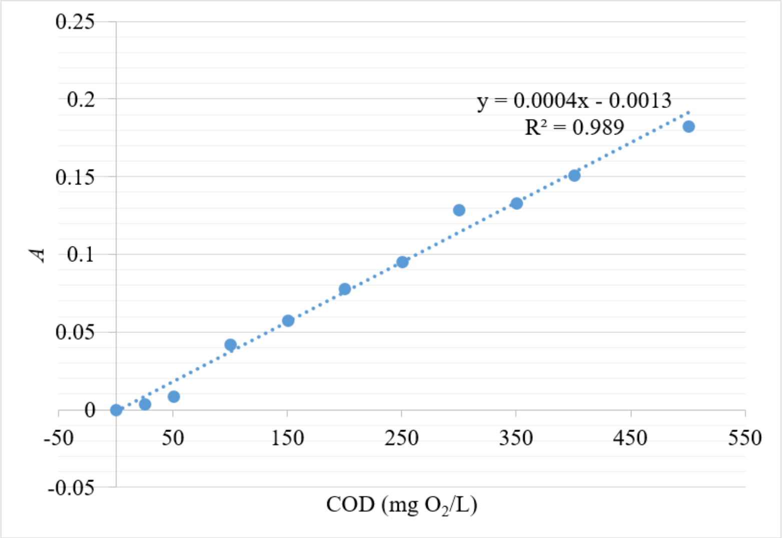

Figure S1. Calibration curve for determination of COD

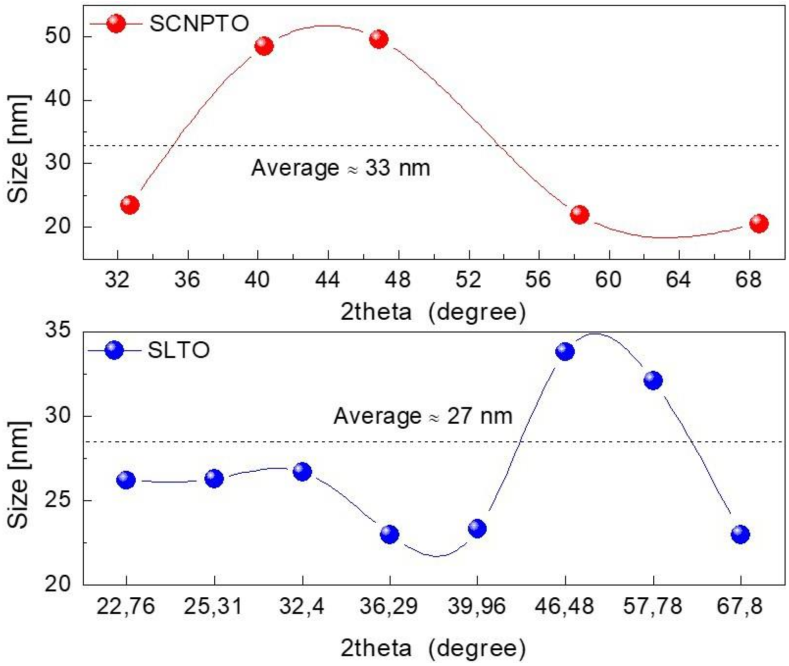

Figure S2. Crystallite sizes as estimated integral values for each peak position calculated for SCNPTO and SLTO

Results and Discussion

Table S1. EDS results for SLTO and SCNPTO nanopowder samples

$Sr_{0.9}La_{0.1}TiO_3$ (SLTO)		$Sr_{0.25}Ca_{0.25}Na_{0.25}Pr_{0.25}TiO_3$ (SCNPTO)	
**Element**	**Atomic %**	**Element**	**Atomic %**
O	68.58	O	60.97
Ti	16.09	Na	4.09
Sr	14.02	Ca	4.04
La	1.32	Ti	22.34
		Sr	4.37
		Pr	4.18

Table S2. Comparison of the experimental and calculated values for SLTO and SCNPTO

	SLTO	SCNPTO
Experimental band gap (eV)	3.50	3.39
Calculated band gap (eV)	3.05	2.67

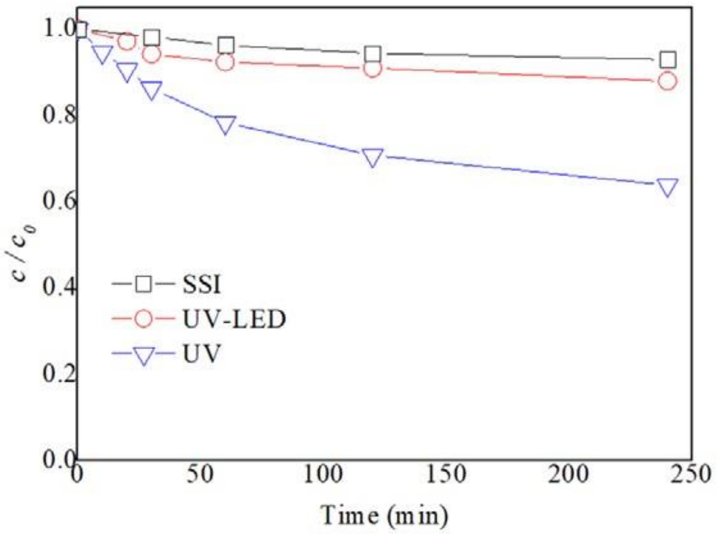

Figure S3. Kinetics of direct photolysis of pindolol (0.05 mmol/dm^3) under different types of irradiation

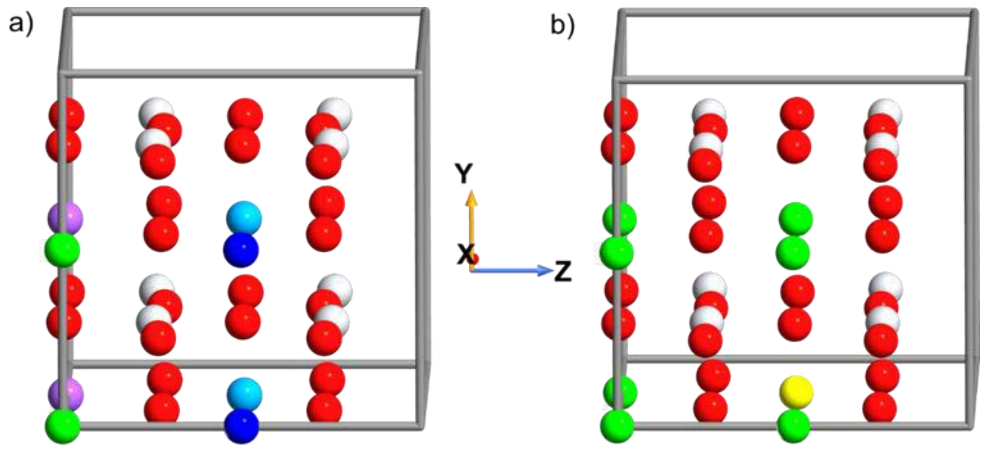

Figure S4. Optimized structures of a) $Sr0._{25}Ca_{0.25}Na_{0.25}Pr_{0.25}TiO_3$ and b) $Sr_{0.9}La_{0.1}TiO_3$
Color coding: oxygen is red, titanium is gray, strontium is green, calcium is dark blue, praseodymium is light blue, and sodium is purple
